# Brain functional network changes in patients with juvenile myoclonic epilepsy: a study based on graph theory and Granger causality analysis

**DOI:** 10.3389/fnins.2024.1363255

**Published:** 2024-05-07

**Authors:** Ming Ke, Yaru Hou, Li Zhang, Guangyao Liu

**Affiliations:** ^1^School of Computer and Communication, Lanzhou University of Technology, Lanzhou, China; ^2^Hospital of Lanzhou University of Technology, Lanzhou University of Technology, Lanzhou, China; ^3^Department of Magnetic Resonance, Lanzhou University Second Hospital, Lanzhou, China

**Keywords:** resting-state functional magnetic resonance imaging, juvenile myoclonic epilepsy, functional connectivity, graph-theory analysis, Granger causality analysis

## Abstract

Many resting-state functional magnetic resonance imaging (rs-fMRI) studies have shown that the brain networks are disrupted in adolescent patients with juvenile myoclonic epilepsy (JME). However, previous studies have mainly focused on investigating brain connectivity disruptions from the perspective of static functional connections, overlooking the dynamic causal characteristics between brain network connections. In our study involving 37 JME patients and 35 Healthy Controls (HC), we utilized rs-fMRI to construct whole-brain functional connectivity network. By applying graph theory, we delved into the altered topological structures of the brain functional connectivity network in JME patients and identified abnormal regions as key regions of interest (ROIs). A novel aspect of our research was the application of a combined approach using the sliding window technique and Granger causality analysis (GCA). This method allowed us to delve into the dynamic causal relationships between these ROIs and uncover the intricate patterns of dynamic effective connectivity (DEC) that pervade various brain functional networks. Graph theory analysis revealed significant deviations in JME patients, characterized by abnormal increases or decreases in metrics such as nodal betweenness centrality, degree centrality, and efficiency. These findings underscore the presence of widespread disruptions in the topological features of the brain. Further, clustering analysis of the time series data from abnormal brain regions distinguished two distinct states indicative of DEC patterns: a state of strong connectivity at a lower frequency (State 1) and a state of weak connectivity at a higher frequency (State 2). Notably, both states were associated with connectivity abnormalities across different ROIs, suggesting the disruption of local properties within the brain functional connectivity network and the existence of widespread multi-functional brain functional networks damage in JME patients. Our findings elucidate significant disruptions in the local properties of whole-brain functional connectivity network in patients with JME, revealing causal impairments across multiple functional networks. These findings collectively suggest that JME is a generalized epilepsy with localized abnormalities. Such insights highlight the intricate network dysfunctions characteristic of JME, thereby enriching our understanding of its pathophysiological features.

## 1 Introduction

Our brain is a network of numerous brain regions where information is continuously processed and transmitted between structurally and functionally connected areas. Studying the human brain as a network of interacting regions can provide new insights into large-scale neurotransmission in the brain. Research has found that the development of most neurological and psychiatric disorders is associated with impaired interconnections between neurons and synapses (Lehrer, 2009). Many brain disorders, such as Alzheimer’s disease (Greicius et al., 2004), schizophrenia (Liu et al., 2008), autism (Kennedy and Courchesne, 2008), attention-deficit/hyperactivity disorder (Tian et al., 2006) and epilepsy (Bettus et al., 2009), often present abnormalities in brain networks. Therefore, the study of brain networks helps us to gain a deeper understanding of the structure and function of the human brain. It also provides a scientific basis for understanding the pathogenesis of neurological and psychiatric disorders, as well as for the prevention, diagnosis and treatment of these disorders.

Juvenile myoclonic epilepsy (JME) is a common type of epilepsy syndrome in adolescents, accounting for 5%–10% of all epilepsy cases. It is also a common type of Idiopathic generalized epilepsy (IGE), making up approximately 26% of all IGE cases (Walsh et al., 2014). The neuropsychological assessment of JME patients revealed a decline in cognitive functioning (Chawla et al., 2021), with impairments observed in tasks related to working memory, prospective memory, decision-making, and other cognitive functions (Wolf et al., 2015). Functional networks can assist in the non-invasive assessment of pathological and physiological changes in epilepsy syndromes and in analyzing the underlying causes of cognitive impairments (Garcia-Ramos et al., 2021). With the advancement of resting-state functional magnetic resonance imaging (rs-fMRI) technology and the maturity of brain network research, researchers have made significant progress in analyzing the pathogenesis of JME. These progresses include a deeper understanding of the brain’s functional network connectivity in epilepsy, focusing on exploring both functional connectivity (FC) and effective connectivity (EC) within these networks. FC measurements can detect coherent spontaneous neuronal activities within a brain network (van den Heuvel and Hulshoff Pol, 2010). EC is a particular type of directionally-related functional connectivity based on statistical models and is used to explain the influence of one neural system on another (Friston, 2011). Studying both aspects helps us understand how pathological processes lead to neural damage.

The whole-brain functional connectivity analysis method is a data-driven approach that constructs a network of functional connections between different brain regions by calculating their correlations. Such methodologies have proven instrumental in uncovering functional connectivity aberrations within specific areas. For instance, the middle temporal gyrus, superior temporal sulcus, and medial thalamus in autism patients (Cheng et al., 2015). This evidence underscores the brain’s integrative nature, where the seamless execution of tasks necessitates a collaborative interplay among various regions. Building on this foundation, graph theory analysis emerges as a pivotal tool for delving into the whole-brain connectome’s topological attributes at a macroscopic scale. Whether in a resting state or engaged in cognitive activity, graph theory can help to nuance how brain networks are organized and adapted over time, including alterations associated with psychiatric disorders, which provides valuable insights into the complex network of neural interactions and the underlying mechanisms of neuropsychiatric disorders. Recent research advances have further solidified the role of graph theory in neurological research. Specifically, graph theory has been applied as a quantitative analysis method in epilepsy (Jiang et al., 2017). Lee et al. (2020) combined graph theory analysis, concluding that there are network abnormalities in the thalamus of JME patients. Despite the growing reliance on functional connectivity magnetic resonance imaging as a formidable technique for mapping large-scale brain networks, critiques regarding its limitations have emerged (Buckner et al., 2013). This highlights the need for a comprehensive approach in neuroscience research, focusing on both the topological properties of functional connectivity networks and the causal dynamics within disease-related brain regions. Based on this, methods for assessing effective connectivity are clearly indispensable. Such thorough investigation is crucial for advancing our understanding of neural mechanisms and improving strategies for disease prevention and treatment.

The effective connectivity methods reflect the dynamic information flow processes between functional brain modules and the interactions between different brain regions. Many studies have supported this using a similar technique to perform brain network assessments and diagnose brain diseases. Using rs-fMRI, researchers conducted a practical connectivity analysis to examine the related changes of idiopathic generalized epilepsy within the major neurocognitive brain networks (Wei et al., 2016). However, it is well known that functional interactions in the brain are highly dynamic rather than static (Lurie et al., 2020). As a new method, dynamic effective connectivity (DEC) is more suitable for studying the directed spontaneous spatiotemporal reorganization of neuronal activity, which is a crucial source of brain fluid dynamics (Zarghami and Friston, 2020). It effectively addresses the issue of previous dynamic studies that only characterize functional connectivity FC based on the temporal correlation between brain signals, thus ignoring the causal influence between brain regions (Friston, 2011). This approach may provide more robust evidence for diagnosing, prognosis, and treating neurological and psychiatric disorders. Researchers used the Granger causality analysis (GCA)-based DEC method and found changes in the default mode network in patients with juvenile myoclonic epilepsy (Zhang et al., 2020). Combining FC and DEC methods to study the aberrant brain functional networks in JME patients is undoubtedly meaningful for neuroscience and psychiatry.

The main purpose of this study was to investigate the differences in the topological properties of brain networks between Healthy Controls (HC) and JME patients, as well as the differences in causal effects between abnormal brain regions. To this end, we first constructed a whole-brain functional connectivity network of the subjects. Then, we calculated the global and nodal properties of the brain networks and identified abnormal brain regions based on the nodal properties. Subsequently, we took the abnormal brain regions in different networks as regions of interest and extracted the time series of the regions of interest for dynamic effective connectivity analysis. We performed dynamic analysis by sliding window method, after which K-means clustering was used to obtain two effective connectivity states, and finally GCA was used to characterize the abnormal causal links between the respective brain regions in the two different states. By this method, we can further understand the abnormal activity of the dynamic brain in JME patients. This analysis provides new perspectives for understanding seizures and cognitive deficits in JME patients.

## 2 Materials and methods

### 2.1 Participants

Our study included 72 participants, with 37 JME patients recruited from the Epilepsy Center of the Second Hospital of Lanzhou University and 35 HC recruited from the local community. The diagnosis of JME was based on the classification criteria for epilepsy of the International League Against Epilepsy (ILAE; Engel, 2001). A routine MRI scan is normal, and routine scalp EEG shows 4–6 Hz generalized spike–wave discharges (GSWDs). Patients are excluded if they have the following features: (1) history of taking antiepileptic drugs, (2) other neurological or psychiatric diseases, (3) other developmental disorders such as autism and intellectual disability, (4) acute physical illnesses that affect the scan. The National Hospital Seizure Severity Scale (NHS3) score is usually used to measure the severity of epileptic seizures. This score is mainly related to the objective clinical events of epileptic seizures (O'Donoghue et al., 1996). The subjects were informed of the content and purpose of the study, and all subjects signed an informed consent form. The study was approved by the Medical Ethics Committee of the Second Hospital of Lanzhou University. Specific demographic characteristics are shown in Table 1.

**Table 1:** Demographic and clinical characteristics of the participants.

	JME (*n* = 37)	HC (*n* = 35)	P-value
Age (years)	19.62 ± 7.17	22.20 ± 6.14	0.11^a^
Sex (males/females)	20/17	13/22	0.15^b^
Handedness (right/left)	37/0	35/0	-
Duration of epilepsy (months)	48.97 ± 47.32	-	-
NHS3 total score	6.26 ± 4.63	-	-

^a^Represents a two-sample *t*-test, ^b^represents a chi-square test; the values are presented as mean ± standard deviation. NHS3, national hospital seizure severity scale; JME, juvenile myoclonic epilepsy; HC, healthy control.

### 2.2 Magnetic resonance imaging acquisition and data preprocessing

MRI data was acquired at the Second Hospital of Lanzhou University using a Siemens Verio 3.0 T scanner (Siemens, Erlangen, Germany) with 16 head coils. Participants were instructed to remain still and avoid any specific mental activity during the scan. Additionally, they were asked to keep their eyes closed and relax while wearing noise-canceling earplugs to minimize any interference from the scanner noise. Use echo planar imaging sequence to obtain resting state functional images for each participant and set as follows: repetition time [TR] = 2,000 ms; echo time [TE] = 30 ms; flip angle = 90°; slice thickness = 4 mm; in-plane matrix resolution = 64 × 64; field of view [FOV] = 240 × 240 mm^2^; number of slices = 33; total volume = 200. For anatomical localization and normalization, a high-resolution structural 3D T1-weighted image was obtained using a magnetization-prepared rapid gradient-echo sequence (TR = 1,900 ms; TE = 2.99 ms; flip angle = 90°; slice thickness = 0.9 mm; acquisition matrix = 256 × 256; field of view = 230 × 230 mm^2^).

Data preprocessing was performed using GRETNA software^1^ based on SPM12.^2^ Specifically, the process involves discarding the first 10 functional images, selecting 190 functional images for each subject. Due to the unsynchronized acquisition time of the whole head image data and the impact of head movement, slice timing correction and realignment are needed. We have removed data with movement greater than 3 mm or rotation greater than 3^°^ caused by head movement. Spatial normalization by DARTEL (warping individual functional images to the standard MNI space by applying the transformation matrix that can be derived from registering the final template file), spatial smoothing with a Gaussian kernel (full width at half-maximum of 6 mm), regressing out covariates (white matter, cerebral spinal fluid, and head-motion profiles were removed by multiple regression analysis to avoid noise signals), temporal linear detrending, and temporal bandpass filtering (0.01–0.08 Hz).

### 2.3 Overview of functional connectivity and dynamic effective connectivity analysis

**Figure 1:**
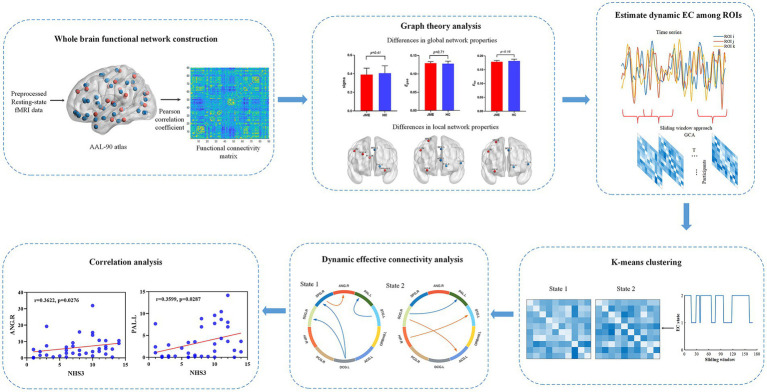
Analysis flowchart.

The analysis workflow of this study is illustrated in Figure 1. Specifically, within this framework, there are six main analysis steps. First, whole-brain functional connectivity analysis is performed on the preprocessed functional data by calculating Pearson correlation coefficients between brain regions to construct the whole network. Secondly, graph theory analysis on the whole-brain functional connectivity network will be performed to compute global and local properties that identify abnormal brain network conditions and regions. Then, using a sliding window approach, the time series of the identified abnormal brain areas are divided into a set of windows, and Granger causality analysis (GCA) is applied to construct causal influence matrices among the ROIs for each window. Afterwards, a k-means clustering method is implemented to cluster all the matrices into discrete EC states, representing transient causal influence patterns during the data acquisition. Subsequently, we evaluated the intergroup differences in the causal influence flow resulting from the abnormal connections between brain regions in specific states for both patient and healthy control groups. Finally, correlations between JME symptom severity and abnormal brain regions and causal influence flow were further assessed.

### 2.4 Whole brain functional network construction

After data preprocessing, the whole-brain functional network was constructed. When constructing a brain network using imaging data, the first step is usually to define nodes and then build edges between them. One standard method to define brain nodes is by using anatomical atlases based on brain structures to define fixed spatially meaningful regions of interest (ROIs). GRETNA software is used to generate brain networks (Wang et al., 2015). Network node definition is as follows: Based on the Automated Anatomical Labeling (AAL) template, the entire brain is divided into 90 cortical and subcortical ROIs, with 45 regions in each hemisphere, each representing a node in network analysis. In the current study, we only focused on these 90 brain regions. The definition of network edge is as follows: The average time series of each ROI for all participants was obtained by averaging the time series of all voxels within the ROI. Subsequently, by calculating the Pearson correlation coefficient between the time series of all possible pairs of brain regions, a 90 × 90 correlation matrix was constructed for each participant. Further, Fisher’s r-to-z transformation is applied to correlation matrices (Liu et al., 2017), converting each correlation matrix into a binarized matrix with sparsity values. When the Pearson correlation coefficient is greater than the sparse value, it is considered that there is a corresponding edge in the brain network (Lu et al., 2017; Wang et al., 2020).

In our study, we applied a sparsity threshold to all correlation matrices ranging from 0.10 to 0.34, with an interval of 0.01. Previous research has emphasized the criteria for selecting the range of sparsity values (Lu et al., 2017). The minimum sparsity was chosen to ensure that the average degree of all nodes in each threshold network was more significant than 2log(N), where *N* = 90 is the number of nodes used in the study. The maximum sparsity was selected to ensure that the small-world scalar (sigma) of each threshold network for all participants exceeded 1.1.

### 2.5 Graph theory analyses

After constructing the whole brain functional network, we computed global and regional brain connectivity measures. We calculated six global metrics to characterize the global properties of the brain functional network: clustering coefficient (C_p_), characteristic path length (L_p_), normalized clustering coefficient (gamma, the ratio of C_p_ between the real and random networks), normalized characteristic path length (lambda, the ratio of L_p_ between real and random networks), global efficiency (E_glob_) and local efficiency (E_loc_). The small-world properties of a network were characterized by gamma and lambda. Typically, a small-world network should meet the following criteria: gamma ≫ 1 and lambda ≈ 1 (Watts and Strogatz, 1998), or sigma = gamma/lambda > 1 (Humphries et al., 2006). Three nodal parameters, node degree centrality, node efficiency, and node betweenness centrality were adopted to describe the nodal properties of the brain functional networks (Achard and Bullmore, 2007). Betweenness centrality is a measure for global efficiency of network topology or resource utilization. So higher betweenness centrality indicates more efficient information flow (Zhou and Lui, 2013). Degree centrality measures a node’s connections with other nodes in the network. A higher degree of centrality means more connections. The node efficiency represents the efficiency of a given node, indicating the efficiency of parallel information transmission in the network. In addition, we computed the area under the curve (AUC) for each network metric. The AUC index has been utilized in previous brain network studies to provide a summarized scalar for brain network topological features independent of individual threshold selection, and it is susceptible sensitive in detecting topological changes related to brain disorders (He et al., 2009; Wang et al., 2009).

### 2.6 Dynamic effective connectivity estimation

#### 2.6.1 Granger causality analysis

After identifying the abnormal brain nodes through graph theory analysis, they were used as regions of interest (ROIs) to extract the time series data of all ROI. In this study, the causal influences among the time courses of ROIs were evaluated using the GCA method. Granger causality estimation assessed the causal effects of the ROIs on other regions (X to Y effect) and the Y to X effect. Unlike other methods of EC measurement, GCA quantifies the causal influence between multiple brain regions in a data-driven manner, without the need to predefine any models (Deshpande and Hu, 2012). Granger causality analysis was conducted using the MATLAB toolbox DynamicBC.^3^ Granger causality is usually used for fMRI data analysis through vector autoregressive modeling, and in GCA, the expression of the joint autoregressive model for the time series Yt and Xt is as follows (Eqs 1, 2):


(1) $$Y_{t}=\overset{p}{\underset{k=1}{\sum }}A_{k}X_{\left(t-k\right)}+\overset{p}{\underset{k=1}{\sum }}B_{k}Y_{\left(t-k\right)}+CZ_{t}+E_{t}$$

(2) $$X_{t}=\overset{p}{\underset{k=1}{\sum }}A_{k}^{\prime }Y_{\left(t-k\right)}+\overset{p}{\underset{k=1}{\sum }}B_{k}^{\prime }X_{\left(t-k\right)}+C^{\prime }Z_{t}+E_{t}^{\prime }$$

where $A_{k}$ and $A_{k}^{\prime }$ are signed-path coefficients, $B_{k}$ and $B_{k}^{\prime }$ are autoregression coefficients, $E_{t}$ and $E_{t}^{\prime }$ are residuals, and $Z_{t}$ represents covariates. The time series $\;X_{t}\;$ asignificantly causes the time series $\;Y_{t}\;$ if the signed-path coefficient Akis significantly larger (Zang et al., 2012).

#### 2.6.2 Sliding window approach

The DEC of ROIs was estimated using the most common method for analyzing brain connectivity dynamics in previous studies, i.e., the sliding window method. Our study used a sliding window size of 22 TR (44 s) and a step size of 1 TR. There were 169 windows (190 TRs) per participant throughout the scan. In particular, a window size of 22 TRs was chosen because it has been shown to provide a good cutoff between kinetic detection ability and the quality of correlation matrix estimates (Allen et al., 2014). Using the time course of all ROIs within each window, 169 EC matrices of size *n* × *n* were obtained for each participant, representing the dynamics of EC between ROIs during resting-state data collection. Previous studies have shown that a window size of 30 to 60 s is sufficient to capture fluctuations in rs-fMRI connectivity stably (Preti et al., 2017).

#### 2.6.3 Clustering analysis and dynamic effective connectivity analysis

K-means clustering is used to identify short-term recurring connectivity patterns, which are predicted by a large-scale neural connectivity model. The K-means algorithm is an unsupervised clustering algorithm that is widely used due to its simplicity and accuracy. For a given set of samples, the algorithm partitions the samples into k clusters based on the distance between the samples. The nodes within each cluster are connected as tightly as possible, while the distance between clusters is maximized. Therefore, the objective of the K-means algorithm is to minimize the squared error E (Li et al., 2020) (Eq. 3):


(3) $$E=\overset{k}{\underset{i=1}{\sum }}\underset{x\in C_{i}}{\sum }||x-\mu_{i}|{|}_{2}^{2}$$

where x is all the sample vectors in the sample set, $C_{i}$ is the sample set of samples whose sample vectors belong to the ith category, and $\mu_{i}$ is the mean vector of the cluster, also known as the center of mass, which is an important property in cluster analysis and is usually obtained by calculating the mean of all the points in the cluster. The center of mass matrix $\mu_{i}$ (Jiao et al., 2021) of the data can be obtained by the following expression (Eq. 4):


(4) $$\mu_{i}=\frac{1}{\left|C_{i}\right|}\underset{x\in C_{i}}{\sum }x$$

Since the samples are high-dimensional data, we used the *L*1 distance function (Manhattan distance; Wang et al., 2016) to determine the similarity of the sample data. The smaller the *L*1 distance, the more similar the two data are. The Manhattan distance expression is given below (Eq. 5):


(5) $$c=|x_{i}-x_{j}|+|y_{i}-y_{j}|$$

where c is the Manhattan distance, $x_{i}$ and $y_{i}$ are the coordinate of the node i in the plane, and $x_{j}$ and $y_{j}$ are the coordinate of the node j .

We used the k-means clustering method to cluster all matrices into discrete connectivity states, representing transient patterns of causal influences during the data collection. The Silhouette Coefficient and Calinski-Harabaz index were used to estimate the optimal number of clusters. The Silhouette Coefficient is the most commonly used evaluation metric for clustering algorithms. It is defined for each sample and measures the average distance between the sample and all other points within the same cluster and the average distance between the sample and all points in the next nearest cluster. The Calinski-Harabaz index, on the other hand, measures the ratio between the separation and compactness of the dataset. It calculates the separation of the dataset by measuring the sum of squared distances between each class center point and the dataset’s center point, and it measures the compactness of the data by summing the squared distances between each point and its class center. Finally, under the determined two connectivity states, we analyzed the causal influence flow of abnormal brain network nodes and compared them between the patient and control groups. We calculated two weighted degree measures, including in-weighted degree and out-weighted degree, the most commonly used measures of causal influence flow (Stevens et al., 2009). In each state, we computed the in-weighted degree measure for a node, which is the sum of the strength of influence from any other node to that node. Additionally, we defined the out-weighted degree measure for a node as the sum of the influence strength from that node to any other node.

### 2.7 Statistical analysis

For statistical analyses of demographic and clinical characteristics between the two groups, the Chi-square test was used for gender and the two-sample t-test for age. To compare the topological properties of the functional brain network (including small-world properties, network efficiency, and node parameters) between JME patients and HC, a series of two-sample t-tests were performed for all parameters within a predefined range of sparsity thresholds (from 0.10 to 0.34). Similarly, two-sample t-tests were conducted for between-group differences in the DEC parameters, which consisted of the flow of causal influences between ROIs in a given state. In addition, Spearman’s correlation analysis was applied to investigate the relationship between abnormal brain topological properties and changes in DEC parameters and clinical characteristics (including duration of epilepsy and NHS3 scores) in JME patients. To control for potential confounding effects, we integrated age and sex as covariates in our two-sample T-test analysis. This approach is essential to ensure a more accurate and reliable comparison between the two groups, thereby improving the validity of our findings. Statistical analyses were performed using SPSS 21.0 and corrected for multiple comparisons using a false discovery rate (FDR; *p* < 0.05).

## 3 Results

### 3.1 Intergroup differences in global network properties

**Figure 2:**
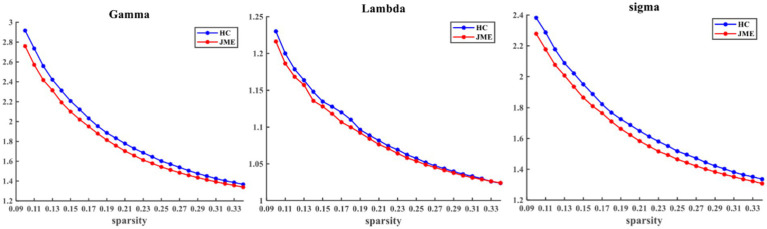
Within the defined threshold range, the normalized clustering coefficient (Gamma) and small-worldness (Sigma) of the JME group and HC group are significantly higher than 1, and the normalized characteristic path length (Lambda) is approximately equal to 1, indicating that all participants meet the typical characteristics of the small-world index. JME, juvenile myoclonic epilepsy; HC, healthy controls.

**Figure 3:**
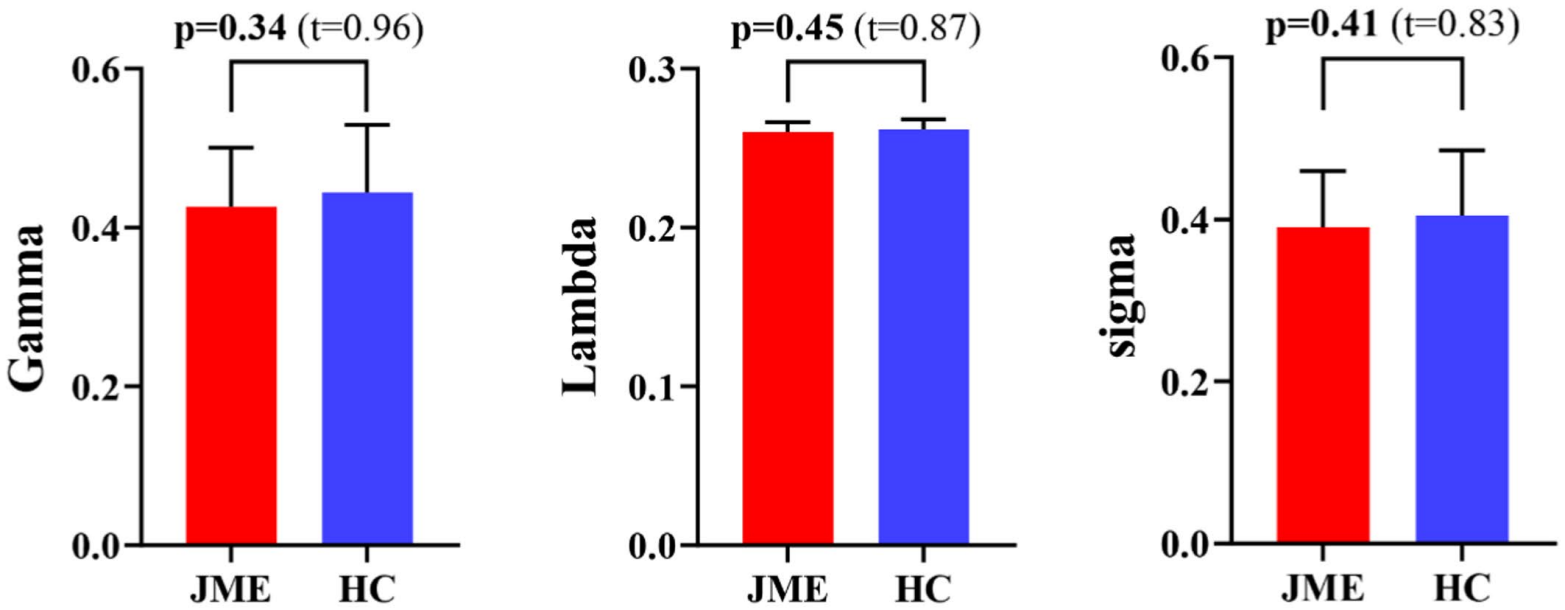
Differences in small-world metrics (Gamma, Lambda, and Sigma) of brain functional networks between JME and HC groups. Error bars represent standard errors. JME, juvenile myoclonic epilepsy; HC, healthy controls.

**Figure 4:**
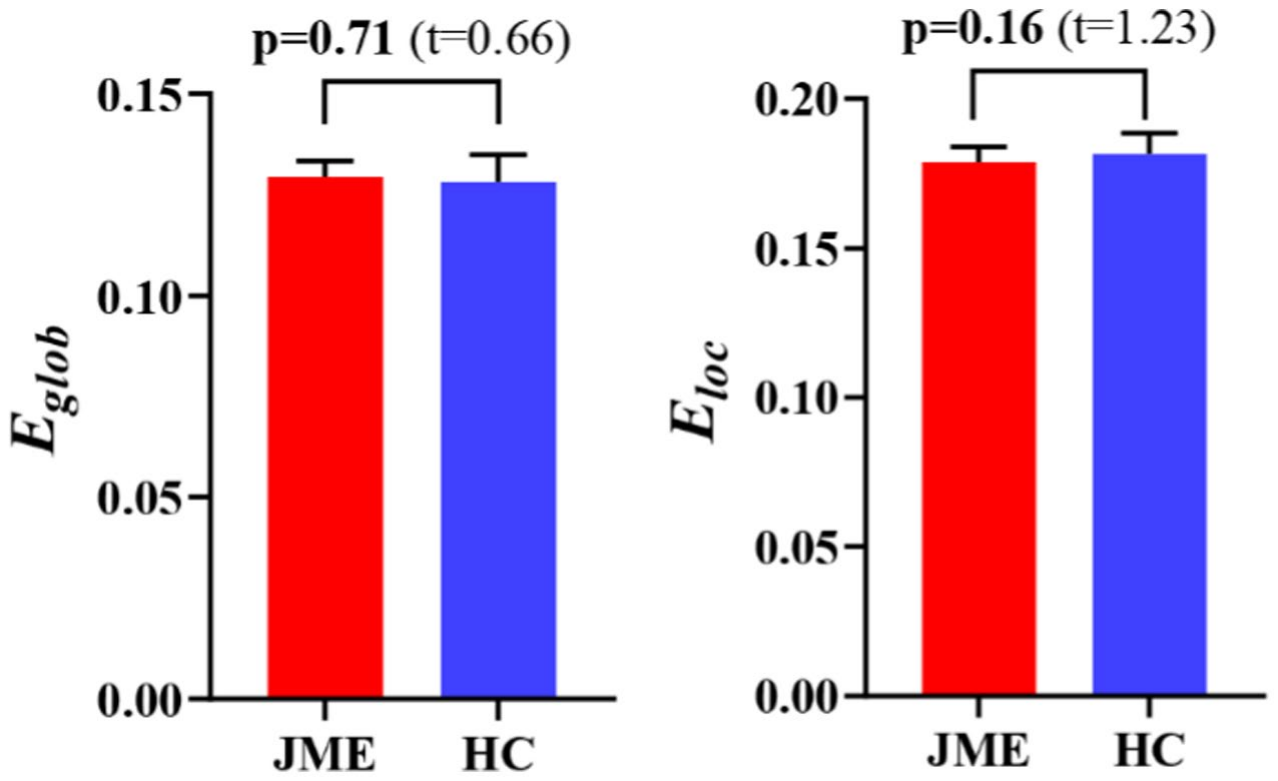
The difference in network efficiency between JME and HC groups. The error bars represent standard error. JME, juvenile myoclonic epilepsy; HC, healthy controls. E_glob_, global efficiency; E_loc_, local efficiency.

At the defined threshold level, the brain functional networks of all participants exhibit significantly higher clustering coefficients (gamma > 1) and nearly identical characteristic path lengths (lambda ≈ 1) compared to comparable random networks. The results demonstrate that all participants in this study possess typical small-world characteristics (sigma = gamma/lambda > 1; Figure 2). The two data groups did not show significant differences in the small-world parameter in the two-sample t-test (Figure 3). Under the given sparsity conditions, there were no significant changes in the statistical comparison of global efficiency (E_glob_) and local efficiency (E_loc_) between the JME patient group and the healthy control group (Figure 4).

### 3.2 Intergroup differences in regional network properties

**Figure 5:**
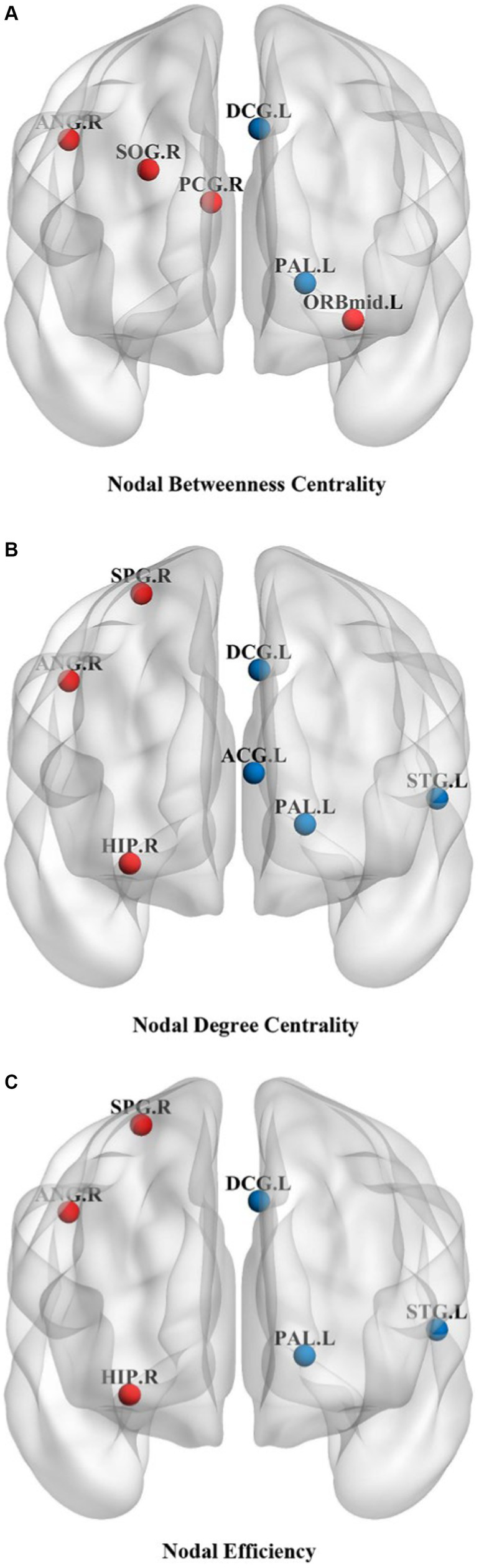
Brain regions showed significant differences in node betweenness centrality **(A)**, node degree centrality **(B)**, and node efficiency **(C)** in specific brain regions between the JME group and HC groups. The red sphere indicates that the JME group’s node attributes have improved compared to the HC group. The blue sphere represents a relative decrease in node attributes of the JME group compared to the HC group. Abbreviations are shown in Table 2.

**Figure 6:**
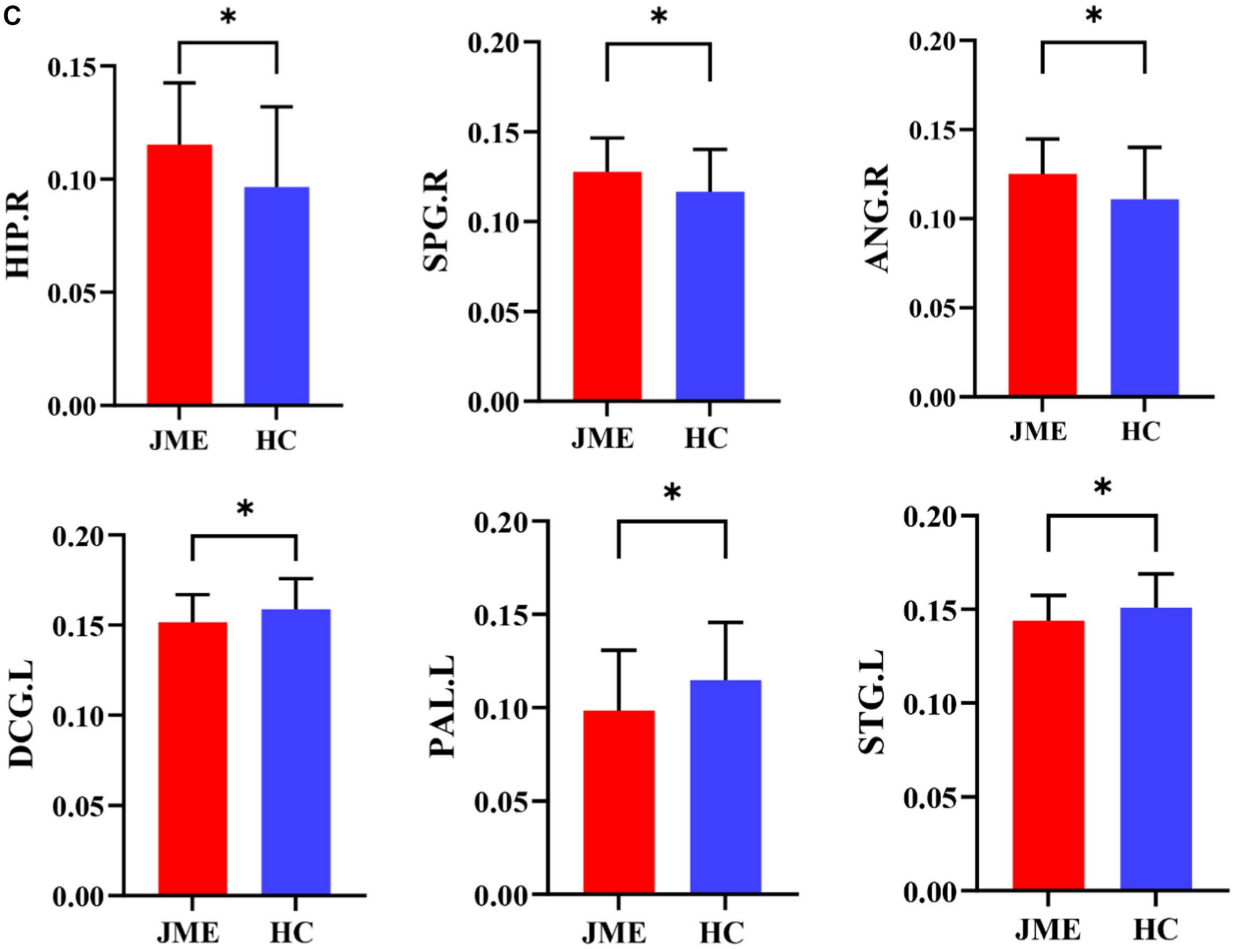
Brain regions showed significant differences in node betweenness centrality **(A)**, node degree centrality **(B)**, and node efficiency **(C)** in specific brain regions between the JME group and HC groups. Error bars represent standard errors. JME: juvenile myoclonic epilepsy; HC: healthy controls, ^*^p < 0.05, ^***^p < 0.01, FDR-corrected.

Changes in node properties (node betweenness centrality, node degree centrality, and node efficiency) were found in multiple brain regions of JME patients (*p* < 0.05, FDR corrected; Figure 5; Table 2). Compared with the healthy control group, the node betweenness centrality of left Median cingulate and paracingulate gyri (DCG.L) and left Lenticular nucleus, pallidum (PAL.L) is decreased in JME patients. In contrast, the node betweenness centrality of the left Middle frontal gyrus, orbital part (ORBmid.L), right Posterior cingulate gyrus (PCG.R), right Superior occipital gyrus (SOG.R), and right Angular gyrus (ANG.R) is increased (Figures 5A, 6A). Meanwhile, the node degree centrality of left Anterior cingulate and paracingulate gyri (ACG.L), left Median cingulate and paracingulate gyri (DCG.L), left Lenticular nucleus, pallidum (PAL.L), and left Superior temporal gyrus (STG.L) is decreased in JME patients. In contrast, the node degree centrality of the right Hippocampus (HIP.R), right Superior parietal gyrus (SPG.R), and right Angular gyrus (ANG.R) is increased (Figures 5B, 6B). The node efficiency of the left Median cingulate and paracingulate gyri (DCG.L), left Lenticular nucleus, pallidum (PAL.L), and left Superior temporal gyrus (STG.L) is decreased. In contrast, the node efficiency of the right Hippocampus (HIP.R), right Superior parietal gyrus (SPG.R), and right Angular gyrus (ANG.R) is increased (Figures 5C, 6C; please refer to Table 2 for the corresponding brain regions).

**Table 2:** Brain regions with abnormal node network characteristics displayed between JME and HC groups.

Brain regions		*P*/*t-*values		
		Nodal betweenness centrality	Nodal degree centrality	Nodal efficiency
**JME > HC**				
	ORBmid.L	**0.04**/**2.05**	0.36/0.93	0.34/1.10
	PCG.R	**0.04/2.21**	0.86/0.18	0.77/0.28
	HIP.R	0.06/1.89	**0.02/2.59**	**0.01/3.02**
	SOG.R	**0.03/2.75**	0.51/0.75	0.44/0.88
	SPG.R	0.27/0.38	**0.03/2.25**	**0.02/2.21**
	ANG.R	**0.04/2.19**	**0.02/2.33**	**0.03/2.28**
**JME < HC**				
	ACG.L	0.53/0.06	**0.04/2.00**	0.10/1.51
	DCG.L	**0.04/2.05**	**0.03/2.31**	**0.04/2.19**
	PAL.L	**0.00/3.33**	**0.02/2.59**	**0.02/2.18**
	STG.L	0.31/0.96	**0.02/2.29**	**0.03/2.09**

Brain regions showing significant between-group differences (*P* < 0.05, FDR corrected, shown in bold font) in at least one of the three nodal network properties are exhibited in the table. HC, healthy controls; JME, Juvenile myoclonic epilepsy patients; R, Right; L, Left; ORBmid, Middle frontal gyrus, orbital part; PCG, Posterior cingulate gyrus; HIP, Hippocampus; SOG, Superior occipital gyrus; SPG, Superior parietal gyrus; ANG, Angular gyrus; ACG, Anterior cingulate and paracingulate gyri; DCG, Median cingulate and paracingulate gyri; PAL, Lenticular nucleus, pallidum; STG, Superior temporal gyrus.

Figures 5, 6 show that the ANG.R demonstrates increased characteristics in terms of node betweenness centrality, degree centrality, and efficiency. This suggests that ANG.R is an essential region for increased network properties. DCG.L and PAL.L are significant regions where the node properties are decreased.

### 3.3 Effective connectivity patterns in dynamic states

**Figure 7:**
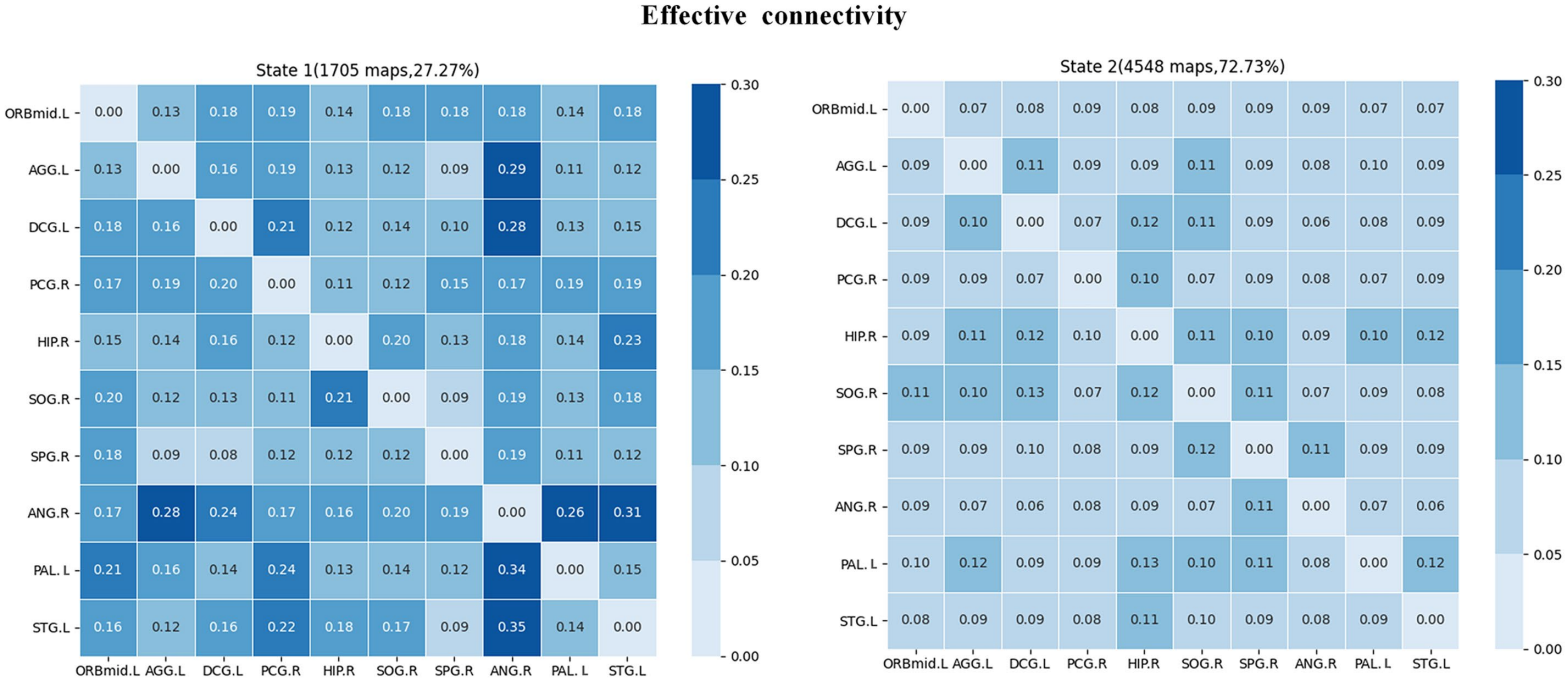
Cluster analysis results of effective connectivity status of JME subjects. The total number and percentage of occurrences are listed above each centroid matrix.

The clustering analysis results for JME patients in each connection state are shown in Figure 7. It can be observed that there are two states in the patient group, and the DEC patterns (cluster centroids) among the ROIs differ significantly between these two states. Specifically, in state 1, the DEC mode exhibits strong mutual influence but with a lower overall occurrence rate (27.27%). In contrast, in state 2, the DEC mode exhibits weak mutual influence but with a higher overall occurrence rate (72.73%). Each matrix represents the cluster’s centroid, reflecting the data’s effective connection state and the number and percentage of occurrences.

### 3.4 Causal impact flow for specific states

**Figure 8:**
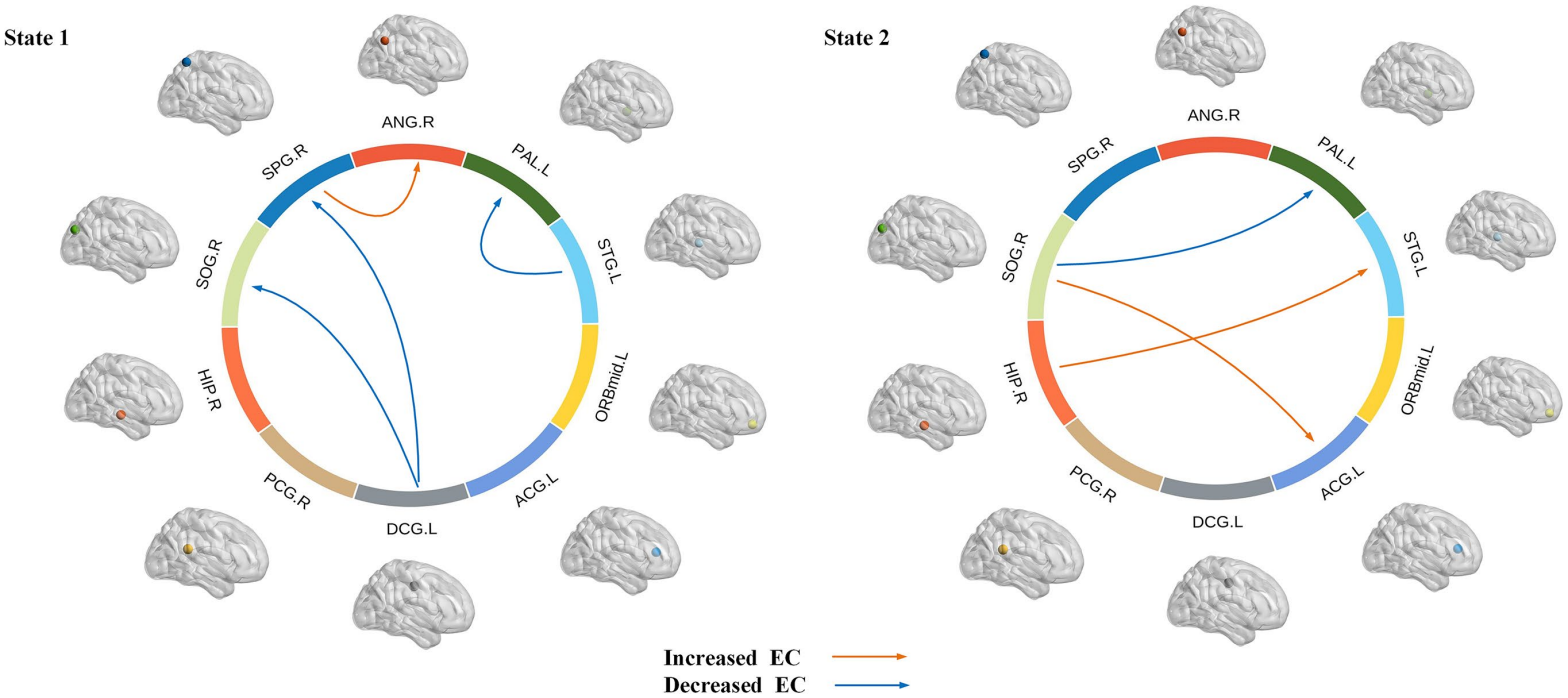
Dynamic effective connectivity modes of different states, where the arrow indicates the direction of causal influence (*p* < 0.05, FDR corrected). Warm and cool colored lines, respectively, represent the increase and decrease of effective connectivity (EC) in JME compared to HC.

The intergroup differences in causal influence flow between ROIs in each state are shown in Figure 8. In state 1, JME patients showed increased EC from SPG.R to ANG.R, and decreased EC from STG.L to PAL.L, DCG.L to SOG.R, and DCG.L to SPG.R compared to the HC group. In state 2, JME patients showed increased EC from SOG.R to ACG.L and HIP.R to STG.L, and decreased EC from SOG.R to PAL.L compared to HC group (*p* < 0.05, FDR corrected). We have also observed that in state 1, these changes in EC are located between the sensorimotor network (SMN), the attention network (AN), the subcortical regions, and the visual network (VN), with the SMN and subcortical regions playing a regulatory role between the networks. In state 2, the changes in EC are located between the subcortical regions, SMN, VN, and the default mode network (DMN), with the VN playing an important role.

### 3.5 Relationship with clinical disease severity

**Figure 9:**
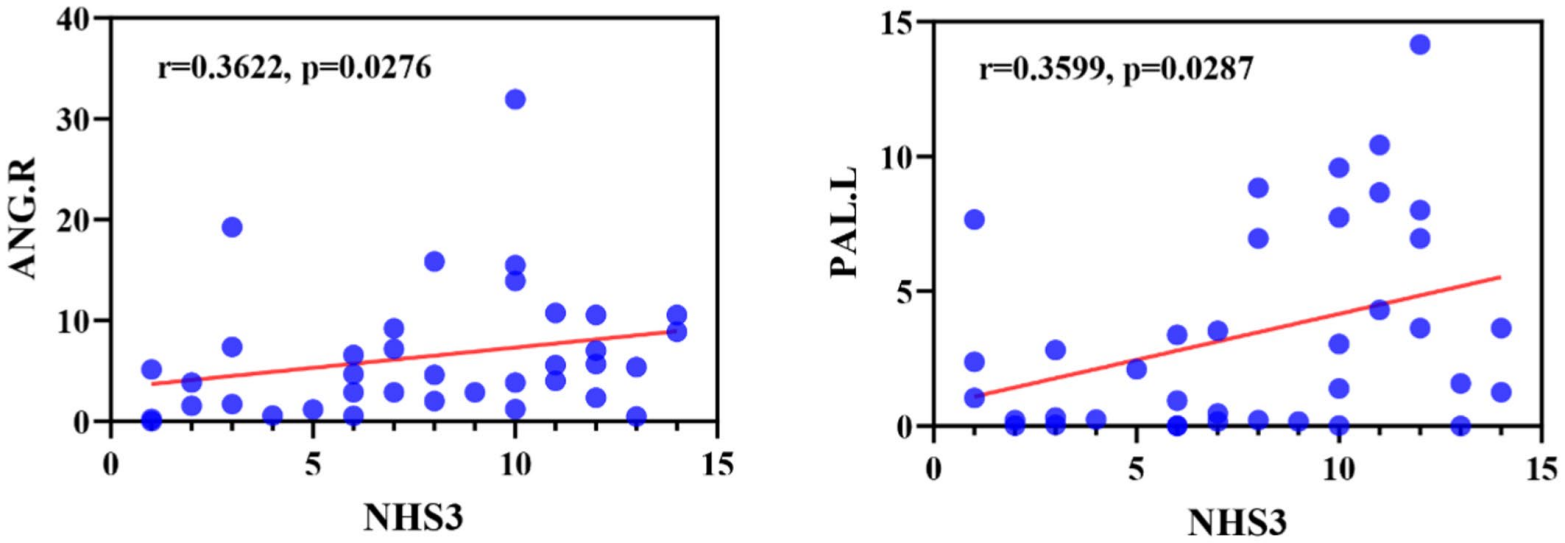
Correlation between NHS3 scores and abnormal brain regions in JME patients in terms of node betweenness centrality. ANG, Angular gyrus; PAL, Lenticular nucleus, pallidum.

We first examined the correlation between abnormal brain regions and clinical characteristics (including duration of epilepsy and NHS3 score), as shown in Figure 9, we found that node betweenness centrality of ANG.R was significantly positively correlated with NHS3 (r = 0.3622, *p* = 0.0276, FDR-corrected), and node betweenness centrality of PAL.L was significantly positively correlated with NHS3 (r = 0.3599, *p* = 0.0287, FDR corrected). No significant correlation was found between duration of epilepsy and abnormal brain regions. In addition, we found no significant correlation between either clinical characteristic and DEC parameters.

## 4 Discussion

The human brain is a complex and interconnected network known for its efficient small-world structure, featuring high local clustering and short path length (Achard et al., 2006). The current study found that the brain networks of JME patients still exhibit small-world characteristics. Previous neuroimaging studies in epilepsy also found that patients with idiopathic generalized epilepsy or temporal lobe epilepsy demonstrated a small-world property of the functional and structural networks (Liao et al., 2010; Zhang et al., 2011). This means that despite abnormalities in neuronal activity, the organizational structure of the brain network in epileptic patients remains relatively stable. Global efficiency reflects the overall information transfer efficiency of the entire network, focusing on the overall information transfer capability. While local efficiency reflects the communication efficiency between neighboring nodes after a node is removed, focusing on the local communication capability after the node is disconnected. Compared with HC, our study did not find significant changes in the overall and local efficiencies of brain networks in JME patients. This suggests that the overall and local information transfer efficiencies in the brains of JME patients remain relatively stable. This is consistent with previous research. When studying the large-scale brain structural network in JME, researchers found no differences in global network characteristics between the two patient groups (Caeyenberghs et al., 2015). These results suggest that the brain functional networks of JME patients do not differ significantly from those of healthy individuals and still possess efficient information processing capabilities.

Concerning node property analysis, the decrease in node properties associated with JME primarily occurred in the paralimbic regions (ACG.L, DCG.L), basal ganglia (PAL.L), and temporal lobe (STG.L). On the other hand, the increase in nodal network properties associated with JME primarily occurred in the frontal lobe (ORBmid.L), paralimbic regions (PCG.R, HIP.R), occipital lobe (SOG.R), and parietal lobes (SPG.R, ANG.R). Some brain regions, known as network hubs, play a core role in supporting the integration of brain network functions, which are involved in various psychiatric and neurological disorders. Research has investigated the developmental processes of core network hubs in the prenatal, infancy, childhood, and adolescence periods using graph theory measures of node centrality in brain networks. It has been found that during adolescence, core network hubs are widely distributed in the frontal lobe, temporal, and subcortical regions. Damage to these core hubs may significantly impact brain network function (Winding et al., 2023). The abnormalities at the regional level in the brain networks of JME patients suggest that the information transmission and integration of nodes throughout the network are disrupted.

The temporal pole is part of the parahippocampal area and is a transitional region from the surrounding cortex to the neocortex (Munoz-Lopez et al., 2010). It is functionally connected to the hippocampus and adjacent neocortex and is involved in the occurrence of epilepsy (Liu et al., 2016). In studies using fMRI to investigate resting-state brain networks, the temporal pole is considered part of the auditory network and is involved in auditory processing (Damoiseaux et al., 2006). We found that the node efficiency of STG.L was reduced in JME patients, which may be the main reason for the abnormalities of the auditory network and the impact on auditory function in patients. The cingulate gyri are located in the medial pericallosal region of each frontal lobe. Due to their diffusely projecting connectivity, the cingulate gyrus plays a crucial role in seizure propagation. A literature search of published cases of tonic–clonic seizures showed that 5 cases originated in the cingulate gyrus (Pearce, 2004). This is consistent with our findings of abnormalities in ACG and PCG. DCG.L is part of the cingulate gyrus and is involved in behavior, motor, and somatosensory functions, especially in emotion, information transmission, and cognitive processing (Oane et al., 2020). Voxel-based morphometric analysis revealed that changes in gray matter volume occurred in DCG.L in patients with JME, which could lead to impaired cognitive function in these patients (Kazis et al., 2021). Node efficiency quantitatively describes the importance of a node in the whole network, the higher the node efficiency, the more important the node is and the more likely it is to become a hub node. Comparative analysis of node efficiencies revealed that in JME, the regions with increased node efficiencies were located in the SPG.R and ANG.R of the parietal lobe. This suggests that the increased importance of these two regions in the functional network of the brain in JME makes them more likely to become hub nodes. This finding is consistent with the findings of Vollmar et al. (2011). Wang et al. (2014) found that the node parameters of ANG.R and HIP.R were altered in TLE patients by studying the topological properties of the whole-brain functional network in temporal lobe epilepsy (TLE) patients. This is consistent with our results. The hippocampus and angular gyrus play crucial roles in memory storage and emotion regulation, and changes in these parameters may be associated with memory deficits in epilepsy patients.

Our study observed nodal characterization anomalies in an important component of the basal ganglia, PAL.L. Combining functional magnetic resonance imaging (MRI) and diffusion tensor imaging (DTI) analysis, significant reductions in gray matter volume and increased mean diffusivity (MD) were found in PAL.L and bilateral HIP of JME patients. These findings further support the notion that macrostructural and microstructural abnormalities in JME are not limited to the thalamus but also affect the basal ganglia and hippocampus. This provides further support for the pathophysiological hypothesis of JME involving the striatum-thalamus-frontal network and suggests disease progression (Kim et al., 2018). The occipital lobe is associated with photosensitive properties in JME patients, especially idiopathic occipital lobe epilepsy (Chilosi et al., 2006). Existing studies show that 30% of JME patients are photosensitive (Wolf and Goosses, 1986). As an essential component of the occipital lobe, the right superior occipital gyrus is involved in higher-level visual associative activity. Our results support the abnormal structural and functional properties of the abovementioned nodes. The orbitofrontal gyrus plays a crucial role in emotion regulation and cognitive control. Our study found an increased betweenness centrality in the ORBmid.L, which plays a crucial role in emotion regulation and cognitive control. It has been suggested that the onset of discharge in patients with JME is not bilaterally synchronized in some sense and that there is a limited focal cortical network for the onset and propagation of discharge, which mainly includes the frontal and temporal cortex, with the orbital frontal gyrus being particularly critical in the frontal cortex. In Holmes’ study of 10 patients with JME, the orbital frontal gyrus was consistently identified in the region of painful discharges across all patients, and notably, in 5 of these cases, it was also situated in the medial floor of the temporal lobe. This observation is consistent with the findings of Zhou (2021), who suggests that there are different frontotemporal thalamocortical networks implicated in patients with JME. Furthermore, it indicates the presence of focal cortical areas that modulate thalamocortical circuits during epileptic seizures, thereby underscoring the critical role of the orbital frontal gyrus in activating epileptic discharge networks in individuals with JME (Holmes et al., 2010).

From an EC perspective, the current work identifies two distinct dynamic states: a less frequent state characterized by strongly connected interactions between regions of interest (state 1) and a more frequent state characterized by weakly connected interactions between regions of interest (state 2). The human brain is increasingly viewed as a dynamic neural system whose functionality depends on different connections between brain regions. It has been indicated that dynamic functional connectivity may reflect the abnormal hypoactive state of epilepsy patients and certain aspects of neural system functional capacity (Kucyi et al., 2017; Zhou, 2021). Our findings and other dynamic state studies of psychiatric disorders, raise the importance of assessing transient aspects of connectivity (Damaraju et al., 2014; Yu et al., 2015). Brain connectivity is highly variable over time, which may represent the flexibility of functional coordination between different brain systems. By performing dynamic functional connectivity analysis on independent components belonging to different functional networks in Parkinson’s disease patients, two states were identified: a sparse state and a dense state. This suggests that different states can reflect different aspects of neural system function (Kim et al., 2017). Zhang et al. (2020) found two distinct connectivity states within the DMN in JME patients through their study on the DEC. They proposed that State 1 may represent an internal-oriented state that supports internally constructed representations, while State 2 may represent an external-oriented state that supports externally constrained representations. In our study, significant reductions in effective connectivity were found in both states compared to healthy controls, especially in state 1, where abnormalities in effective connectivity occurred between multiple cortical regions such as temporal, basal, limbic, and parietal lobes, which can lead to reduced information transfer and coordination between these regions, and this reduction in effective connectivity may have contributed to the more pronounced low brain in state 1 in patients. Activity. In addition, we found that in state 1, changes in effective connectivity were particularly associated with the three identified regions of significant abnormality. The decrease in effective connectivity was also significant compared to state 2. This suggests that state 1 exhibits more regions of significant abnormal firing and abnormal neuronal inhibition compared to state 2. The characteristics of state 1 can be considered as a potential early warning signal for seizures. Therefore, monitoring and analyzing different brain states is beneficial for early diagnosis.

Anomalous causal links in the two states were found to exist between multiple cortical regions, particularly in the parietal lobe (right ANG, right SPG), the occipital lobe (right SOG), the limbic lobe (left DCG, right HIP, left ACG), and the basal ganglia (left PAL), they are located within different brain networks. These regions located within the basal ganglia network, VN, SMN and DMN, are important structures in the brain. They play a crucial role in motor control, emotional regulation, and cognitive processes, among other functions. Cognitive impairments and increased psychopathological risk in epilepsy may be attributed to disrupted causal relationships among core neurocognitive brain networks (Wei et al., 2016). The ANG and SPG are important areas of the parietal lobe, and the SPG plays a pivotal role in many sensory and cognitive processes, including motor sensory, visuospatial attention and socially relevant behavioral control. The ANG is associated with the processing of visual information and visual perception. It is involved in the discrimination, recognition and categorization of visual objects and in regulating spatial perception and attention. We found enhanced effective connectivity from the SPG.R to the ANG.R in our patients, which may imply increased information transfer between the SPG.R and the ANG.R and may contribute to more effective visual information processing and regulation of visual perception. The STG.L is the main region of the temporal lobe. Lee et al. (2014) explored the causal influence between cortical regions in JME patients by analyzing EEG data. They found that during the slow-wave descent phase, patients exhibited maximal outflow from the temporal cortex, and that reduced effective connectivity from STG.L to PAL.L in state 1 may be the main cause of maximal outflow from the temporal cortex. Similarly, alterations in effective connectivity in the hippocampus, temporal lobe, and prefrontal cortex are critical for understanding seizures. Previous studies based on mouse animal models have shown extensive cortical and subcortical functional network abnormalities in focal hippocampal seizures (Englot et al., 2008). The temporal lobe is generally associated with language, auditory, memory, and emotional-affective functions. And its particular function for language is interpretation and presentation in addition to language comprehension, that also contributes to social cognition and emotional processing. The hippocampus and temporal lobe are closely related. In state 2, we found that the connections from HIP.R to STG.L were enhanced. This result validates the theory that abnormal hippocampal activity and epileptic discharges are likely to affect the temporal lobe (Chassoux et al., 2004; Ritchey et al., 2015).

In addition, ample evidence suggests that multiple brain network functions are impaired in JME patients, including subcortical networks, DMN, SMN etc. (Zhong et al., 2018; Lee and Park, 2019). Jiang et al. (2018) studied the functional and causal connectivity of the AN and DMN in patients with refractory epilepsy. They found that frequent seizures in patients with refractory epilepsy may impair the cortex, disrupting the AN and DMN networks and leading to changes in functional and causal connectivity. Additionally, epileptic activity may disrupt network interactions and affect information exchange. The study found that multiple brain network nodes in JME patients have abnormal causal connections, indicating that seizures are not just a localized brain region problem but a systemic issue involving the entire brain network. This provides significance for the diagnosis and treatment of the disease.

Finally, this study also found a significant correlation between the node betweenness centrality with significant anomalies and NHS3 scores. The angular gyrus is involved in social cognition and language, and given its rich connectivity and location of multisensory information convergence, the angular gyrus resembles a cross-modal integrative hub (Seghier, 2013). The pallidum is located in the basal ganglia-thalamus-cortex neural network and is a central region for the induction of myoclonic seizures (Huh et al., 2018). Through our analysis, we found that betweenness centrality in the angular gyrus and pallidum was significantly and positively correlated with NHS3 scores, speculating that the angular gyrus and pallidum may serve as potential targets for epilepsy therapy, with the potential to improve symptom control in patients with epilepsy by intervening in the functional activity or network connectivity of these regions. Similarly it has been shown that an increase in the number of high betweenness centrality in a population of patients with epilepsy serves as a marker of enhanced connectivity, and is similarly effective in identifying those patients who are more likely to have persistent seizures (Grobelny et al., 2018).

Our research is subject to the following limitations: Firstly, our sample size needs to be increased, and further analysis of the connectivity abnormalities between brain networks should be conducted in a larger population of JME patients. Secondly, some studies have found that there are differences in susceptibility to epilepsy between males and females (Savic, 2014). In the future, we will consider gender in investigating differences in brain functional networks between males and females with JME. Thirdly, a rough division of the whole brain into 90 regions based on the AAL template was used to construct functional brain networks. A previous study showed that different division schemes may lead to different results in graph theory metrics (Zalesky et al., 2010). In future studies we will choose a finer template, which will not only help to identify and extract regions of interest more accurately, but also explore the functional connectivity and interactions between different brain regions in more depth. Furthermore, although this research is dedicated to exploring brain network properties, our construction of network models has not directly accounted for white matter functionality. White matter functioning is indeed a significant part of brain networks. A body of research has shown that BOLD signals can be used to infer white matter function (Ji et al., 2017, 2019, 2023). Ignoring white matter function may potentially restrict the depth of our comprehension of the overall dynamics and functional aspects of brain networks. Therefore, it is imperative for future studies to introduce and effectively integrate white matter functional data into brain network models in order to improve their accuracy and thoroughness.

## Data availability statement

The raw data supporting the conclusions of this article will be made available by the authors, without undue reservation.

## Ethics statement

The studies involving human participants were reviewed and approved by the Epilepsy Center of Lanzhou University Second Hospital. Written informed consent to participate in this study was provided by the participants’ legal guardian/next of kin. Written informed consent was obtained from the individual(s), and minor(s)’ legal guardian/next of kin, for the publication of any potentially identifiable images or data included in this article.

## Author contributions

MK: Methodology, Writing – original draft, Writing – review & editing. YH: Methodology, Writing – original draft, Writing – review & editing. LZ: Data curation, Writing – review & editing. GL: Data curation, Writing – review & editing.

## Footnotes

^1^www.nitrc.org/projects/gretna/

^2^http://www.fil.ion.ucl.ac.uk/spm

^3^www.restfmri.net/forum/DynamicBC
